# Unexplained Tachypneoa and Severe Metabolic Acidosis in a Three-Month-Old Child: A Rare Presentation of Beta-Ketothiolose Deficiency

**DOI:** 10.7759/cureus.21934

**Published:** 2022-02-05

**Authors:** Vijayakumary Thadchanamoorthy, Kavinda Dayasiri

**Affiliations:** 1 Clinical Sciences Department, Faculty of Health Care Sciences, Eastern University, Batticaloa, LKA; 2 Pediatrics, Faculty of Medicine, University of Kelaniya, Colombo, LKA

**Keywords:** mitochondrial acetoacetyl-coa thiolase, 2-methyl-3-hydroxybutyrate, metabolic acidosis, carnitine, ketones, isoleucine, beta-ketothiolase deficiency

## Abstract

Beta-ketothiolase (mitochondrial acetoacetyl-CoA thiolase, T2) deficiency is a rare inborn error of metabolism that is characterized by impaired metabolism of ketones and isoleucine. The condition is inherited as an autosomal recessive disorder. Herein, we present a child with T2 deficiency from Mahaoya, Eastern Province, Sri Lanka. This three-month-old child presented with fever, difficulty in breathing, and irritability for one day and was subsequently found to have severe metabolic acidosis with positive ketone bodies in urine. His blood glucose was normal. Metabolic screening showed increased urinary excretion of 2-methyl-3-hydroxybutyrate (2M3HB), 2-methyl acetoacetate (2MAA), and tiglylglycine (TIG). He was diagnosed to have beta-ketothiolase deficiency based on biochemical studies. Genetic studies were not done due to financial constraints in the family. Severe metabolic acidosis was successfully managed with intravenous sodium bicarbonate infusion. T2 deficiency would be a differential diagnosis in children with unsolved ketoacidosis. Children with T2 deficiency have a better outcome if detected and managed early. The reported patient had age-appropriate growth and development at the latest follow-up at three years eight months while he has been on oral carnitine and bicarbonate.

## Introduction

Beta-ketothiolase deficiency is an autosomal recessive disease characterized by a defect in acetoacetyl-CoA thiolase, which is a mitochondrial enzyme. This condition was initially described by Daum et al. in 1971 [1]. The disorder occurs secondary to a pathogenic mutation in the ACAT1 gene, which affects isoleucine catabolism and ketone body utilization [1-2]. The ACAT1 gene is sited at chromosome 11q22.3-23.1 in humans and has over-reported 70 mutations to date. Most children with a T2 deficiency first present within the first two years of life and the episodes are usually precipitated by infection, fasting, and protein-rich meals. Subsequently, the frequency of episodes declines with increasing age. One of the cardinal laboratory findings is increased urinary excretion of isoleucine-catabolic intermediates 2M3HB, 2MAA, and TIG. In addition, serum acylcarnitine analysis reveals elevated tiglylcarnitine (C5:1) and 2-methyl-3-hydroxy-butyryl-carnitine (C5-OH) levels [2]. However, few T2-deficient patients reveal unusual clinical and biochemical results. The gravity of the manifestations with regard to severity and rate of occurrence differs among children with this condition. The prognosis is generally favorable unless acute severe ketoacidosis attacks result in irrevocable sequelae [1,3].

## Case presentation

The index child is a three-month-old boy who was second born to non-consanguineous parents from Eastern Sri Lanka. He was delivered at full term weighing 3 kg. He had been on starter infant formula since birth. There was no history of neonatal death in the family. His elder sister was five years old and healthy. The child had grown age-appropriately till three months and his weight was 4.8 kg at the time of admission to the local hospital. He presented with fever, difficulty in breathing, and irritability for one day. On examination, he was ill, drowsy, dehydrated, tachypneic, and crackles were noted in both lung fields. The rest of the system examinations had been clinically normal. Subsequently, he was transferred to the medical intensive care unit (MICU) of the regional tertiary care hospital in Eastern Sri Lanka.

Full blood count showed leukocytosis (WBC - 24.37 × 10^3^/μl, N-65.8), mild anemia (hemoglobin - 10.9 g/dL), and platelets were 425x10^3^/μl. C-reactive protein was less than 6 mg/dL and erythrocyte sedimentation rate was 20 mm/first hour. Arterial blood gas showed severe metabolic acidosis with respiratory compensation: pH - 7.0604, pO2 - 88.5 mmHg, pCO2 - 8.2 mmHg, HCO3 - 2.3 mmol/L, and base excess (BE) - 20. Ketonuria was detected. The rest of the investigations revealed blood glucose of 164 mg/dL, lactate of 1.1 mmol/L, and ammonia of 145 μmol/L. His renal and liver functions had been normal. The 2-D echocardiogram was normal. Tall T waves were seen in the ECG recording on the multi-para monitor and were confirmed by a 12-lead ECG rhythm strip. The electroencephalogram revealed an age-appropriate normal brain electrical activity. He was initially identified to have a possible metabolic disorder characterized by severe metabolic acidosis, which was likely precipitated by a lower respiratory tract infection. He was commenced on intravenous cefotaxime empirically, and paracetamol suppositories were given for the fever in addition to correction of dehydration and acidosis. Formula milk was continued. Investigations over the period of the hospital stay are listed in Tables 1-2.

**Table 1:** Investigations reports while in the MICU and ward WBC, white blood cell; PCV, packed cell volume; ESR, erythrocyte sedimentation rate; CRP, C-reactive protein; AST, aspartate transaminase; ALT, alanine transaminase; INR, international normalized ratio; APPT, activated partial thromboplastin time; LDH, lactate dehydrogenase; ECG, electrocardiograph, MICU, medical intensive care unit

Investigations	Value on Day 1 of MICU	Value on discharged from the ward	Reference range
WBC	22.3X10^3^	10.2X10^3^	4-10X10^3^
Neutrophil	65.8%	55.2%	
Lymphocytes	32%	38%	
PCV	45%	35%	32-35
Haemoglobin	10.9g/dL	10 g/dL	13-14
Platetlet	425x10^3^	225x10^3^xg/dL	150-450
ESR	20mm /hour	20 mm/hour	<20
CRP	<6 mg/dL	<6 mg/dL	<6
Procalcitonin	0.4 ng/ml	0.5 ng/ml	0.5
Peripheral blood smear	No abnormal cells, normochromic normocytic picture		
Serum albumin	3.5 g/l	3.1 g/l	34 – 50
AST	40 U/L	35 U/L	10 - 40
ALT	35 U/L	35 U/L	10 - 40
Random blood glucose	164 mg/dl	98 mg/dl	80-120
Serum bilirubin - total	20 µmol/l	15 µmol/l	3- 20
Serum bilirubin - direct	1 µmol/l	1 µmol/l	<3
Serum ammonia	145 μmol/L	120 μmol/L	68-136
Serum lactate	1.1mmol/L	0.8 mmol/L	1-3.3
Serum calcium	9.8 mg /dL	10.2 mg /dL	8-10
Serum magnesium	1.3 meq/L	1.5 meq/L	1.4-1.7
Prothrombin time	15 Seconds	14 Seconds	10 - 14
INR	1.1	1.2	2-2.2
APTT	30 Seconds	25 seconds	25 -35
Serum fibrinogen	180 mg/dL		200 to 400
D-dimer	0.2 mg/L	< 0.25 mg/L	< 0.3
Serum triglycerides	154 mg/dL		< 150
Serum LDH	-	245 U/L	140 – 280
Serum sodium	138 meq/l	140 meq/l	135 - 145
Serum potassium	5.5 meq/l	4.1 meq/l	3.5-5.1
Blood urea	60 mg/dl	45 g/dl	
Serum creatinine	0.8	1	0.5-1.2
Serum ferritin	325	325	24-336
Serum amylase	85 u/dL	90 U/L	30-110 U/L
Serum lipase	80u/dL	86U/L	0-160U/L
Urine for ketones	Detected	Not detected	
Blood cultures	No growth isolated		
Urine cultures	No growth isolated		
ECG	Tall ‘T’ wave (multipara monitor)	Normal	
Chest X-ray and echocardiogram	Normal		
Ultrasound brain and abdomen	Normal		

**Table 2:** Blood gas reports while in the MICU and ward MICU, medical intensive care unit

Day	pH	HCO_3_	pO2 (mmHg)	pCO2 (mmHg)	Base Excess (BE)
Day-1	7.15	6	98	20	18
Day-2	7.28	10	100	28	14
Day-3	7.3	18	115	32	8
Day-7	7.4	26	90	41	0.9
Day -12	7.22	18	85	28	6
Day -15	7. 35	24	115	30	4
Day -30	7.34	23	95	35	4

As the child became drowsier, and his level of consciousness deteriorated, he was subsequently ventilated. Since oral intake was low, he was managed symptomatically with 10% intravenous glucose-containing fluids and electrolytes and intravenous bicarbonate. As the initial pH was less than 7.1 ( pH-7.0604) and the child had adequate hemodynamic stability, he was given NaHCO3 - 0.5 mmol/kg over 20 minutes, followed by continuous infusion. Bicarbonate infusion and ventilatory support were adjusted every six hours with arterial blood gases. Since the child responded on Day 2 both biochemically and clinically, bicarbonate infusion was tailed off and omitted on Day 3. The possible infective causes, such as severe bronchiolitis, septicemia, meningoencephalitis, and bronchopneumonia, had been excluded by subsequent investigations including negative urine and blood cultures. Lumbar puncture was not performed, as his parents did not consent. Since tracheal culture and PCR also revealed no organisms, his intravenous antibiotics, cefotaxime and clarithromycin, were stopped after seven days. All possible metabolic disorders presenting with acidosis were considered including most of the aminoacidurias whilst symptomatic management was offered to the patient. Finally, an acylcarnitine analysis of dried blood spots revealed an elevated C5:1 (0.34 μmol/L; cut-off point 0.12) and C5-OH (2.68 μmol/L; cut-off point 0.47) levels. Urine analysis revealed very high ketones, which also included grossly elevated 3 hydroxybutyric (3HB) and acetoacetic acids (AcAc) and mild elevation of 2M3HB, 2MAA, and TIG. Based on the findings of these investigations, he was diagnosed to have T2 deficiency. His parents were counseled and educated to avoid prolonged fasting. Modified rice-based milk was prescribed to prevent protein overloading via nasogastric feeding following assessment by the nutritionist. Carnitine (100 mg/kg/day) supplementation was also given. A dried blood spot was preserved from the patient and parents for ACAT1 mutational analysis. He was discharged with oral bicarbonate and carnitine after five weeks of hospital stay. Arrangements were made to follow up in the clinic.

The child has currently been on monthly follow-up with basic investigations and arterial blood gas analysis. The child has been thriving well with age-appropriate development. He had two mild breakthrough attacks following viral fever and acute gastroenteritis. These episodes were managed with supportive care, including intravenous fluids containing adequate dextrose, anti-emetics, and oral probiotics. Oral carnitine and bicarbonate were adjusted based on arterial blood gases. Monitoring of blood carnitine level was not done due to the unavailability of the system in the present hospital. The child recovered promptly and was discharged in three days during each episode. Parent education regarding the disease and early detection of ominous signs greatly impacted this child’s timely management, and, currently, the child is three years and eight months old with appropriate physical growth and development. Parents follow counseling and are waiting to have another child once their genetic studies have been performed.

## Discussion

**Figure 1:**
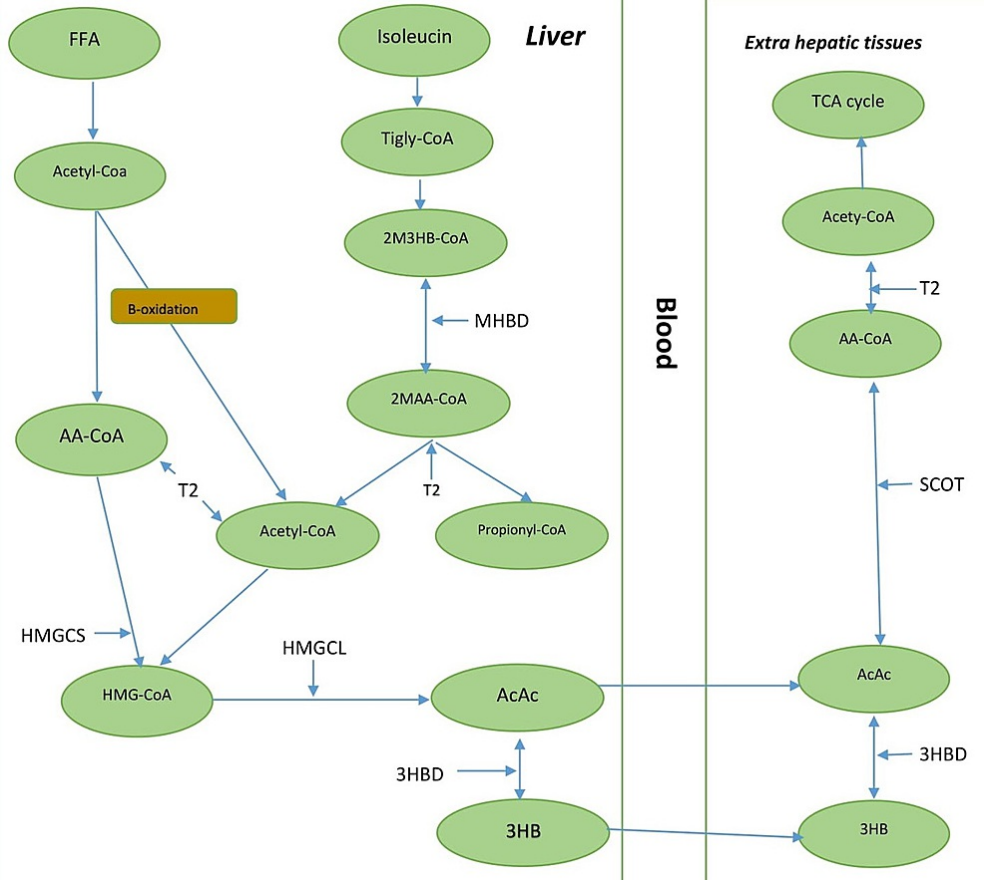
Role of beta-ketothiolase enzyme in the liver and extrahepatic tissues 2M3HB indicates 2-methyl-3-hydroxybutyryl; 2MAA, 2-methylacetoacetyl; 3HB, 3-hydroxybutyrate; 3HBD, 3-hydroxybutyrate dehydrogenase; AA, acetoacetyl; AcAc, acetoacetate; CoA, coenzyme A; FFA, free fatty acids; HMG-CoA, 3-hydroxy-3-methylglutaryl-CoA; HMGCL, HMG-CoA lyase; HMGCS, mitochondrial HMG-CoA synthase; MHBD, 2-methyl-3-hydroxybutyryl-CoA dehydrogenase; SCOT, succinyl-CoA:3-oxoacid CoA transferase; T2, mitochondrial acetoacetyl-CoA thiolase; TCA, tricarboxylic acid

T2 deficiency results in impaired catabolism of isoleucine and utilization of ketone bodies that eventually result in severe ketoacidosis. The disorder is associated with raised 2M3HB, 2MAA, acylcarnitine, and TG in urine [3]. The ketone bodies, such as AcAc and 3HB, are produced in the liver and serve as energy sources for most tissues except for the liver (Figure 1) [4]. The brain utilizes ketones as the main substitute for glucose. Other names for beta-ketothiolase deficiency include T2 deficiency, mitochondrial acetoacetyl-CoA thiolase deficiency, 3-oxothiolase deficiency, 3-ketothiolase deficiency, and 2-methyl-3hydroxybutric academia [5].

T2 deficiency presents in two ways as either classical or atypical. The classical type exhibits clinical manifestations such as frequent ketoacidosis episodes between six months and 18 months. However, most patients are free of symptoms in between these episodes. Ketoacidosis attacks have been precipitated by various factors, which include infections either viral or bacterial, fasting, fever, or other stresses. Possibly, the attack was precipitated by a viral lower respiratory tract infection in the reported child. Severe attacks may lead to coma, convulsions, and sometimes death. Sequelae of severe metabolic decompensation might lead to neurodevelopmental impairment resulting in long-term complications although the overall prognosis is usually good. Some children present with atypical clinical features during very early childhood, especially in the neonatal period. Further, few children may exhibit neurological symptoms before the onset of ketoacidosis attacks. In some settings, early diagnosis is made either following neonatal screening or investigation of an affected family member [6-8].

All patients presenting with ketoacidosis need to be screened for metabolic disorders and acute samples of blood, serum, and urine should be preserved for metabolic assay before treatment is initiated. The child may be discharged once an acute episode is cured although a thorough metabolic evaluation should be performed to arrive at the underlying diagnosis [8-10]. In T2 deficiency, the blood total ketones body (TKB) level during acute episodes must be more than 7 mmol/L and blood sugar and blood ammonia are usually normal. However, hypoglycemia or hyperglycemia may be noted occasionally. Few patients have been reported having mildly elevated ammonia levels [5]. High urinary excretions of 2MAA, 2M3HB, and TIG are evident in this disorder. Blood acylcarnitine assay generally shows an increased C5:1 and C5-OH levels. Genetic studies of the affected children with T2 deficiency demonstrate defects in the ACAT1 gene [1-2].

The management of T2 deficiency is categorized into three stages: 1) treatment of the acute episode, 2) anticipation and prevention of future episodes, 3) screening and counseling of the family members. Conservative treatment with correction of fluid and electrolytes and correction of acidosis, and hypoglycemia (if any) during acute episodes lead to the improvement of the illness [8-10]. Avoidance of prolonged fasting and increased carbohydrate intake during other illnesses should be considered in these patients. Glucose infusion during vomiting and monitoring urinary ketone bodies during illnesses should be done to prevent ketotic episodes. If ketonuria presents, temporary restriction of protein to a certain extent, avoidance of fatty food, and supplementation of L-carnitine are recommended. Asymptomatic family members can be diagnosed by screening with acylcarnitine analysis of dried blood spots and are advised to avoid the risk factors that are known to precipitate acute episodes [1,5]. Screening of newborns for T2 deficiency may reveal false-negative results due to the mild genotypic types [11].

The overall prognosis is generally good when detected and treated early before the development of life-threatening complications. Family counseling is mandatory to prevent ketoacidosis attacks in the affected individuals. Multidisciplinary team care improves the long-term outcomes of these patients.

## Conclusions

This report presents a young male infant who presented with a rare condition known as a beta-ketothiolase deficiency. Diagnosis of this condition can be improved with the introduction of comprehensive newborn screening programs in low-resource settings. Timely diagnosis and management of this condition help avoid acute life-threatening as well as chronic long term unacceptable sequelae and improve the short- and long-term prognosis of these children.
